# Beneficial Roles of Bioconversion-Derived Metabolites of Dietary Components as Potential Biological Regulators in Obesity and Type 2 Diabetes Mellitus

**DOI:** 10.3390/nu18182980

**Published:** 2026-09-11

**Authors:** Heaji Lee, Yunsook Lim

**Affiliations:** 1 Department of Food Science and Nutrition, Hallym University, Chuncheon 24252, Republic of Korea; ji3743@hallym.ac.kr; 2 Department of Food and Nutrition, Kyung Hee University, Seoul 02447, Republic of Korea

**Keywords:** bioconversion, metabolites, obesity, type 2 diabetes, nutritional intervention

## Abstract

Obesity and type 2 diabetes are associated with alterations in gut microbial composition and host nutrient metabolism, both of which can modify the bioconversion of dietary compounds. Consequently, identical dietary substrates may generate distinct metabolite profiles, leading to different physiological responses. Emerging evidence suggests that many biological effects of dietary compounds are mediated, in part, by metabolites generated through host and microbial bioconversion. These metabolites regulate key pathways involved in oxidative stress, inflammatory signaling, glucose metabolism, and gut barrier integrity. Altered bioconversion efficiency observed in obesity and type 2 diabetes may therefore influence metabolic outcomes by altering the quantity and composition of bioactive metabolites. This review summarizes current evidence on the metabolic effects of bioconversion-derived metabolites generated from dietary components and discusses their underlying molecular mechanisms in obesity and type 2 diabetes. By focusing on metabolites generated through dietary bioconversion rather than dietary substrates alone, this review proposes a metabolite-centered framework for understanding variability in dietary responses and identifying metabolically relevant targets for future nutritional interventions.

## 1. Introduction

Obesity and type 2 diabetes are characterized by chronic metabolic dysfunction involving impaired glucose and lipid metabolism, disrupted energy homeostasis, and persistent low-grade inflammation [1,2]. Progressive metabolic abnormalities in these conditions contribute to functional decline across multiple tissues, including adipose tissue, liver, skeletal muscle, and brain [3]. Impaired metabolic flexibility also disrupts metabolic responses to nutrient intake, further aggravating disease progression and increasing the risk of secondary metabolic complications.

As nutritional status strongly influences metabolic regulation under these conditions, nutritional intervention has become an important strategy for the prevention and management of obesity and type 2 diabetes [4,5,6]. However, metabolic responses to dietary interventions vary considerably among individuals, and similar dietary exposures do not always result in comparable metabolic outcomes [7,8]. These observations suggest that the biological effects of diet cannot be explained solely by dietary intake itself and highlight the importance of processes occurring after nutrient ingestion [9,10,11].

Dietary substrates undergo extensive transformation through digestive processes, host enzymatic reactions, and gut microbial metabolism before reaching systemic circulation [11]. Consequently, target tissues are frequently exposed to bioconversion-derived metabolites of dietary components (BDMs) rather than the original dietary compounds. The beneficial effects of dietary factors may be primarily mediated by these metabolites, underscoring their central role in determining dietary efficacy.

Recent evidence suggests that BDMs are not merely secondary products of nutrient metabolism but biologically active molecules that directly regulate metabolic processes in target tissues [12,13,14]. Several metabolites exhibit beneficial biological activities distinct from those of their precursor compounds [12,13]. Therefore, growing attention has been directed toward understanding the role of BDMs as biological regulators linking dietary intake to metabolic health.

In obesity and type 2 diabetes, gut microbial dysbiosis is characterized by reduced microbial diversity and alterations in bacterial taxa involved in bioconversion of dietary substrate, thereby affecting the generation of biologically active metabolites [15,16,17]. For example, reduced abundance of butyrate-producing bacteria, including *Faecalibacterium* and *Roseburia*, is associated with decreased short-chain fatty acid production [18]. Likewise, alterations in bacterial taxa involved in ellagic acid and daidzein metabolism may impair the generation of urolithins and S-equol, respectively [19,20,21]. These changes may contribute to interindividual differences in metabolite availability and biological responses to dietary interventions.

In this review, we focus on BDMs as potential mediators of these effects and examine their metabolic effects in obesity and type 2 diabetes. Interindividual variation may further contribute to differences in metabolic responses to the same dietary components [22,23,24]. Therefore, several approaches have been investigated to modify microbial metabolism and metabolite production, including dietary interventions, probiotics, prebiotics, and synbiotics [25].

However, these strategies often require prolonged intervention to reshape the gut microbiota and frequently produce variable responses among individuals [15,16,17]. BDMs therefore provide a complementary perspective for understanding how differences in post-ingestion bioconversion may contribute to variability in metabolic responses. Direct administration of BDMs may further provide a means of examining metabolite-specific effects independently of their microbial production, although the observed effects may still be influenced by other sources of biological variability [26,27,28,29].

Research directly evaluating individual BDMs in obesity and type 2 diabetes remains limited, with most evidence derived from cellular and animal studies. This reflects the early stage of BDM research and limits conclusions regarding their translational potential. Nevertheless, current findings provide a basis for identifying candidate BDMs and defining the evidence required for their further development. Therefore, this review critically examines the current evidence for individual BDMs and identifies key gaps that need to be addressed for their translation. Future research directions are also proposed for evaluating promising BDMs in obesity and type 2 diabetes.

## 2. Bioconversion-Derived Metabolites of Dietary Compounds (BDMs)

Bioconversion refers to the transformation of dietary substrates into structurally distinct metabolites through host enzymatic processes and gut microbial activity occurring before or after absorption [11,24]. This process determines the quantity and quality of metabolites generated from dietary compounds, thereby influencing the biological effects of dietary compounds.

Current evidence indicates that BDMs may serve as the functional mediators of biological responses [30,31]. In many cases, BDMs exhibit greater systemic availability, stability, and tissue accessibility than the original dietary substrates [32,33]. Ellagic acid, for example, shows limited systemic bioavailability, whereas gut microbiota-derived urolithins are more readily absorbed and persist at substantially higher circulating concentrations [20]. Furthermore, urolithins exhibit biological activities distinct from those of their precursor compound, including the regulation of mitochondrial function and inflammatory signaling [20,26]. Similarly, S-equol, a microbial metabolite generated from the soy isoflavone daidzein, exhibits greater estrogen receptor affinity, antioxidant activity, and anti-inflammatory potential than its precursor compound [34,35,36]. Although direct comparative evidence between precursor compounds and BDMs remains limited, these findings support the possibility that biological responses to dietary compounds may be mediated, at least in part, by downstream metabolites generated through microbial and host metabolic conversion.

Gut microbiota is a major contributor to this bioconversion process, particularly for compounds that are poorly absorbed in the upper gastrointestinal tract [11]. Gut microbial metabolism involves degradation, reduction, deconjugation, and structural modification of dietary polyphenols, fibers, and amino acid derivatives [11,24]. The generation of BDMs is influenced by gut microbial composition because microbial taxa possess distinct enzymatic capacities for substrate utilization and metabolic conversion [24].

Several representative microbiota-dependent bioconversion pathways have been well characterized. Ellagitannin-containing foods such as pomegranate, berries, and walnuts are converted into urolithins through microbial bioconversion pathways involving bacterial taxa including *Gordonibacter urolithinfaciens* and *Gordonibacter pamelaeae* [37]. Dietary fibers are metabolized by short-chain fatty acids (SCFAs)-producing bacterial communities including *Faecalibacterium prausnitzii, Roseburia intestinalis,* and *Eubacterium rectale*, generating metabolites such as butyrate, acetate, and propionate [18]. Microbial metabolism of flavan-3-ols derived from cocoa, tea, and grapes produces phenyl-γ-valerolactones through the sequential activity of specific bacterial taxa, including *Eggerthella lenta, Flavonifractor plautii*, and *Adlercreutzia equolifaciens* [33]. Similarly, soy isoflavones such as daidzein are converted into S-equol by S-equol-producing bacteria including *Slackia isoflavoniconvertens*, *Slackia equolifaciens,* and *Adlercreutzia equolifaciens* [38]. Tryptophan is also transformed into bioactive indole derivatives, including indole-3-propionic acid, by bacterial species such as *Clostridium sporogenes* [39]. Collectively, these examples demonstrate that the generation of BDMs depends on the presence and metabolic activity of specific microbial taxa.

In parallel, host enzymatic processes further modify absorbed substrates and microbial metabolites [11]. These modifications occur during intestinal and first-pass hepatic metabolism through oxidation and conjugation reactions. Consequently, they influence the bioavailability and biological activity of BDMs. Taken together, these findings suggest that BDMs may serve as mediators linking dietary exposure to metabolic responses.

In obesity and type 2 diabetes, host–microbiota interactions and BDM profiles may be altered [15,16,40]. BDM concentrations are further influenced by dietary intake and host metabolic processes, including intestinal absorption and hepatic or renal metabolism, independently of microbial production [15]. Conversely, altered microbial metabolism and BDM availability may contribute to host metabolic dysfunction [15,40]. Therefore, circulating or intestinal BDM levels reflect the combined influence of dietary intake, microbial bioconversion, and host metabolic processes.

Direct administration has been used to investigate the biological effects of individual BDMs by bypassing microbial conversion of dietary precursors. Although this approach reduces variability arising from BDM formation, subsequent responses remain subject to host metabolism and other biological factors [41,42]. Evaluating individual BDMs may therefore provide additional insight into the metabolic effects of dietary bioconversion that cannot be determined from precursor intake alone.

## 3. Key Classes of BDMs Relevant to Obesity and Type 2 Diabetes

Direct administration of BDMs has emerged as a useful approach for investigating biological effects in obesity and type 2 diabetes [42]. Because obesity and type 2 diabetes may impair the conversion of dietary substrates into beneficial BDMs [15], direct supplementation of BDMs provides an opportunity to evaluate metabolite-specific effects independent of bioconversion efficiency. The following sections summarize representative BDMs that have been investigated in experimental models of obesity and type 2 diabetes (Table 1).

### 3.1. Polyphenol-Derived Microbial Metabolites

#### 3.1.1. Urolithins

Urolithins are gut microbiota-derived metabolites generated from ellagitannin-containing foods such as pomegranates, berries, and nuts [12,32]. Conversion of ellagitannins into urolithins depends on specific microbial taxa, including *Gordonibacter urolithinfaciens* and *Gordonibacter pamelaeae* [37]. Therefore, the capacity to produce urolithins differs among individuals according to gut microbial composition.

Among the identified urolithins, urolithin A has been the most extensively investigated because of its effects on mitochondrial function and metabolic regulation. Urolithin A improved mitochondrial health by enhancing mitophagy and mitochondrial quality control in C. elegans and rodents [43]. Improved mitochondrial function may enhance the oxidation of metabolic substrates for energy production, a process frequently impaired in obesity and type 2 diabetes.

In high-fat diet-induced obese mice, urolithin A prevented excessive body weight gain by increasing energy expenditure and adipose tissue thermogenesis [44]. Urolithin A also improved insulin sensitivity and reduced adipose tissue inflammation and hepatic lipid accumulation [26,44]. In high-fat diet/streptozotocin-induced diabetic mice, urolithin A improved fasting blood glucose, glucose tolerance, and pancreatic β-cell function [45].

However, evidence for the metabolic benefits of urolithin A is primarily derived from animal studies. Human urolithin production varies considerably according to gut microbial composition, and differences in urolithin-producing phenotypes can influence urolithin exposure after consumption of ellagitannin-containing foods [42]. Further clinical studies are needed to determine whether the metabolic effects observed in animal models are also evident in humans.

#### 3.1.2. Phenyl-γ-Valerolactones and Related Phenolic Acids

Phenyl-γ-valerolactones are major microbial metabolites generated from flavan-3-ols, such as catechins and procyanidins, and can be further converted into smaller phenolic acids [33]. Their formation involves sequential microbial transformations, and the contribution of individual bacterial taxa may vary according to the microbial community and substrate [33]. Compared with precursor flavan-3-ols, phenyl-γ-valerolactones exhibit greater systemic availability and distinct biological properties [33].

Several downstream metabolites of phenyl-γ-valerolactones have been investigated for their metabolic effects. Treatment with 5-phenylvaleric acid increased glucose oxidation in primary human skeletal muscle cells and enhanced glucose-stimulated insulin secretion in pancreatic β-cells [46]. Related phenolic metabolites have also shown metabolic effects in vivo. In high-fat diet-fed mice, supplementation with 3-(3′,4′-dihydroxyphenyl)propanoic acid and 3′,4′-dihydroxyphenylacetic acid reduced body weight gain and improved insulin sensitivity [47]. Metabolomic analysis further identified changes in metabolites associated with the pentose phosphate pathway and TCA cycle [47]. However, evidence for their metabolic effects in obesity and type 2 diabetes remains limited.

#### 3.1.3. S-Equol

S-Equol is a gut microbiota-derived metabolite generated from the soy isoflavone daidzein through microbial bioconversion by specific intestinal bacterial species [21], including *Slackia isoflavoniconvertens* [38]. Because S-equol production depends on specific intestinal bacteria, the capacity to generate S-equol varies among individuals [35]. Consequently, individuals consuming similar amounts of soy isoflavones may exhibit different circulating S-equol levels depending on their gut microbial composition.

Compared with its precursor daidzein, S-equol exhibits higher affinity for estrogen receptor β and distinct biological activity [34,36]. Equol-producing individuals may also exhibit greater improvements in lipid profiles, blood pressure, vascular function, and other cardiometabolic risk markers following soy isoflavone interventions than non-producers [35]. However, evidence linking equol-producing status to these metabolic benefits remains limited [35].

Direct S-equol supplementation has been investigated as a strategy to bypass microbial bioconversion and interindividual variability in equol production [48]. In Zucker diabetic fatty rats, S-equol supplementation improved glucose tolerance, enhanced insulin secretion, and ameliorated abnormalities in lipid metabolism through modulation of ChREBP/TXNIP signaling [29]. In a randomized placebo-controlled trial, S-equol supplementation improved glycemic control, lipid profiles, and arterial stiffness, with greater improvements observed in participants who were unable to produce equol endogenously [48]. However, evidence from clinical trials remains limited.

Collectively, S-equol may contribute to the metabolic effects associated with soy isoflavones, and direct supplementation may reduce variability associated with microbial S-equol production. Further clinical studies are needed to determine whether these metabolic effects are consistent across populations and metabolic conditions.

### 3.2. Short-Chain Fatty Acids (SCFAs)

SCFAs, including acetate, propionate, and butyrate, are generated through microbial fermentation of dietary fibers by diverse bacterial taxa [18,71]. SCFA production also involves microbial cross-feeding, in which metabolites produced by one bacterial group can be utilized by other bacteria for SCFA production [18,71]. SCFA production varies with dietary fiber intake, gut microbial composition, and microbial activity. Therefore, identical dietary fiber intake may not result in comparable SCFA production across individuals [17,18].

Reduced abundance of SCFA-producing bacteria has frequently been reported in obesity and type 2 diabetes [15,18]. However, reduced bacterial abundance does not necessarily indicate a corresponding reduction in SCFA production. Fecal SCFA concentrations also do not directly reflect SCFA production or systemic exposure because SCFAs undergo extensive intestinal absorption and metabolism [71,72]. The metabolic fate of individual SCFAs also differs. Butyrate is extensively utilized by colonocytes, whereas propionate is largely metabolized in the liver and acetate has greater systemic availability [71]. These differences can influence their metabolic actions across tissues and physiological conditions [71]. The effects of SCFAs may also vary with concentration, receptor signaling, and metabolic context [72].

Butyrate supplementation promotes mitochondrial regulation and fatty acid oxidation through AMPK-related signaling in obese mouse models [49,50]. In skeletal muscle of obese mice, butyrate enhanced mitochondrial function and oxidative metabolism [49,52]. In type 2 diabetic rats, sodium butyrate supplementation reduced hyperglycemia, insulin resistance, dyslipidemia, and hepatic steatosis [51]. These effects were accompanied by enhanced Akt phosphorylation and suppression of FOXO1-mediated hepatic gluconeogenesis [51]. Sodium butyrate is commonly used in experimental studies, although direct administration differs from physiological exposure through colonic fermentation. This difference should be considered when interpreting the metabolic effects of butyrate supplementation in relation to dietary fiber fermentation.

Propionate has been primarily investigated in the context of glucose and lipid metabolism [53,54]. In obese rodents, propionate supplementation reduced hepatic lipogenesis and improved glucose homeostasis [53]. Propionate also regulates intestinal hormone signaling [55,56]. In primary colonic cultures and in vivo rodent models, propionate stimulated GLP-1 and PYY secretion, whereas these responses were attenuated in FFAR2-deficient mice, supporting a role for FFAR2 in intestinal propionate sensing [56]. However, these effects are not consistent across experimental models. In an isolated perfused rat colon, propionate did not consistently stimulate GLP-1 or PYY secretion [57]. These findings indicate that intestinal responses to propionate depend on experimental conditions.

Acetate, the most abundant SCFA in circulation, has also been implicated in the regulation of energy balance and lipid metabolism [58,59,60]. In adipose tissue, acetate-mediated activation of FFAR2 has been linked to reduced insulin signaling and fat accumulation, thereby promoting lipid utilization in peripheral tissues [58]. Acetate may also regulate energy balance through hypothalamic signaling [59]. In mice, acetate derived from colonic fermentation reached the hypothalamus and altered neuronal activity and neuropeptide expression involved in appetite regulation [59]. However, opposite effects have been reported in other rodent models. Increased acetate production in high-fat diet-fed rats activated parasympathetic signaling and increased ghrelin and glucose-stimulated insulin secretion, leading to hyperphagia and weight gain [60]. These contrasting findings suggest that the metabolic effects of acetate may vary across tissues and experimental conditions. The route and source of acetate exposure may also contribute to these differences [72].

Taken together, individual SCFAs exert distinct metabolic effects across target tissues, and their actions may vary with concentration, receptor expression, dose, and metabolic condition [72]. However, the effects of direct SCFA administration should be distinguished from those resulting from dietary fiber fermentation. In addition to SCFA production, fiber fermentation can influence multiple microbial and host processes [71,72].

Despite these differences, current evidence supports the metabolic relevance of individual SCFAs in obesity and type 2 diabetes and suggests their potential as metabolic modulators. Direct administration of specific SCFAs may provide a strategy to utilize these effects while reducing variability associated with microbial production.

### 3.3. Tryptophan-Derived Microbial Metabolites

Tryptophan metabolism generates a broad network of microbial and host-derived metabolites. Gut microbial metabolism produces indole and several indole derivatives, including indole-3-lactic acid (ILA), indole-3-propionic acid (IPA), indole-3-acetic acid (IAA), and indole-3-aldehyde (IAld). Other microbial metabolites, such as tryptamine, are generated from tryptophan. Tryptophan is also metabolized through host pathways, including the kynurenine pathway.

Indole is a major microbial metabolite of tryptophan and has been implicated in intestinal and metabolic regulation [61]. Indole contributes to enteroendocrine signaling [61]. In diabetic mice, indole administration improved glucose tolerance and insulin sensitivity. It also increased intestinal L-cell differentiation and GLP-1 secretion [62]. These findings suggest that indole may influence glucose homeostasis through intestinal signaling.

ILA is another microbial metabolite derived from tryptophan. ILA has been primarily investigated for its role in intestinal barrier and immune regulation [63]. Experimental studies suggest that ILA regulates intestinal barrier integrity and inflammation through aryl hydrocarbon receptor (AhR)-dependent signaling [63]. However, direct evidence for its metabolic effects in obesity and type 2 diabetes remains limited.

IPA is a gut microbiota-derived metabolite generated from dietary tryptophan by microbial taxa including *Clostridium sporogenes* [39]. In prospective observational studies, higher circulating IPA levels were associated with a lower risk of type 2 diabetes [67]. However, these associations do not establish causality and may be influenced by dietary and metabolic factors.

Experimental studies have further demonstrated metabolic effects of direct IPA administration. In high-fat diet-induced obese mice, oral IPA supplementation reduced metabolic dysfunction and improved colonic barrier integrity [64]. These effects were associated with increased intestinal tuft cells and interleukin (IL)-25 production [64]. In high-fat diet/streptozotocin-induced diabetic rats, oral IPA administration reduced fasting glucose and preserved pancreatic islet architecture [65]. IPA also increased PI3K/Akt/glucose transporter 4 (GLUT4) signaling in skeletal muscle [65]. More recently, oral IPA administration preserved pancreatic β-cell function and improved glucose homeostasis in HFD-fed and db/db mice by reducing endoplasmic reticulum (ER) stress through forkhead box A1 (FOXA1)–sphingosine-1-phosphate phosphatase 1 (SGPP1)–heat shock protein family A member 5 (HSPA5) signaling [66].

IAA has also been implicated in metabolic regulation. In high-fat diet-induced obese mice, oral IAA supplementation attenuated body weight gain and hepatic steatosis while improving glucose metabolism [41]. IAA also increased hepatic Gpha2 expression and regulated thyroid-stimulating hormone (TSH)-related pathways involved in lipid metabolism [41].

**Figure 1:**
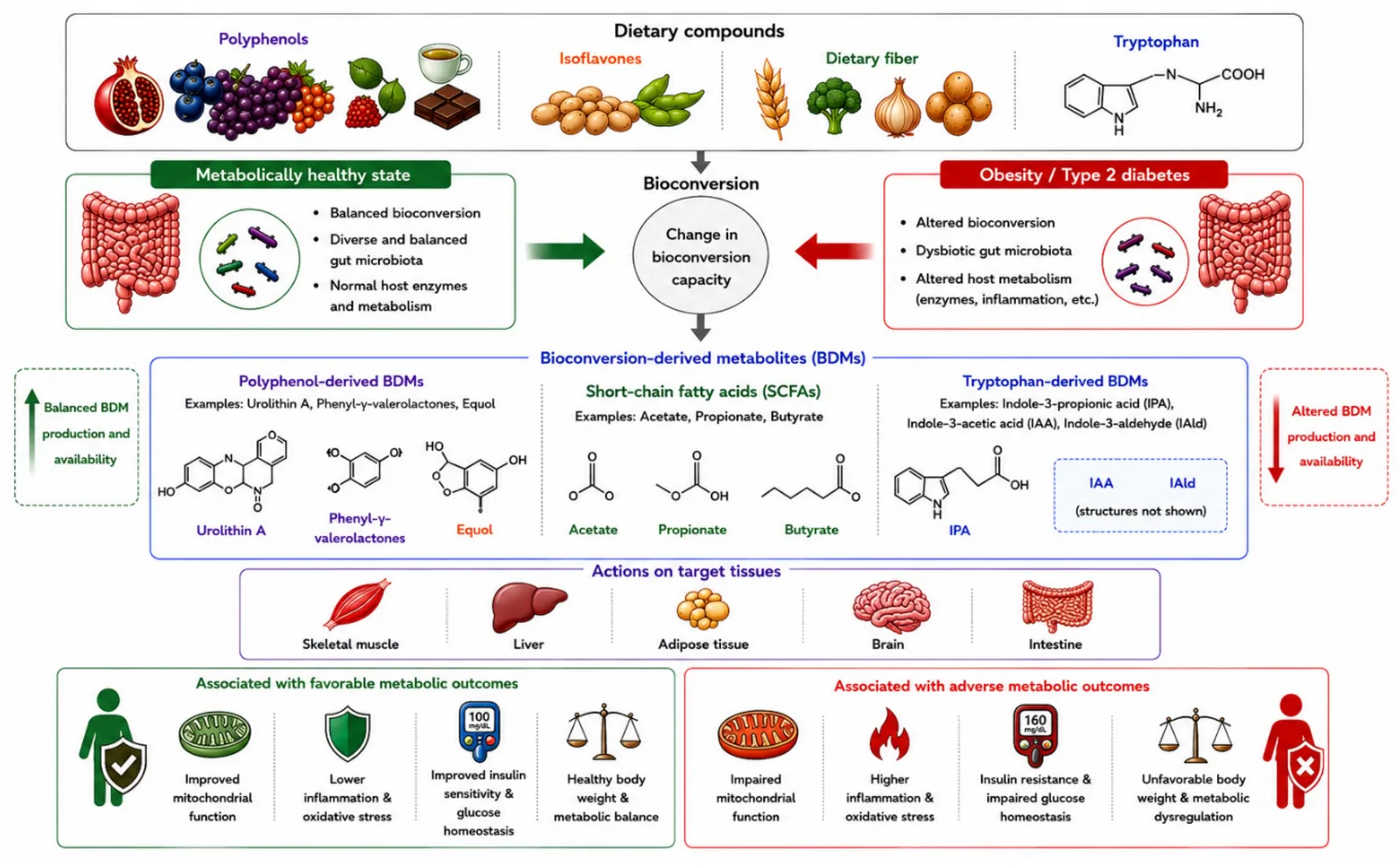
Conceptual framework illustrating changes in bioconversion capacity and bioconversion-derived metabolite profiles in obesity and type 2 diabetes. Dietary compounds, including polyphenols, isoflavones, and tryptophan-containing substrates, undergo host- and microbiota-mediated bioconversion to generate biologically active metabolites such as urolithins, equol, short-chain fatty acids (SCFAs), indole-3-propionic acid (IPA), indole-3-acetic acid (IAA), and phenyl-γ-valerolactones. In metabolically healthy conditions, efficient bioconversion promotes favorable metabolite profiles and robust metabolic responses. In contrast, obesity and type 2 diabetes are associated with altered gut microbiota composition and bioconversion capacity, which may modify metabolite profiles and contribute to variable or attenuated metabolic responses. These metabolites influence mitochondrial function, inflammatory signaling, insulin sensitivity, and overall metabolic homeostasis.

IAld has primarily been investigated for its role in intestinal barrier and immune regulation through AhR signaling [68]. Following ligand binding, AhR translocates to the nucleus and forms a heterodimer with the AhR nuclear translocator (ARNT) [68]. The AhR–ARNT complex binds to xenobiotic response elements and regulates transcriptional programs involved in epithelial and immune responses [69]. In intestinal immune cells, microbial tryptophan metabolite-mediated AhR activation has also been linked to IL-22 production [68]. IL-22 contributes to epithelial defense and barrier integrity. Consistent with its role in intestinal homeostasis, IAld alleviated gut barrier disruption and promoted intestinal stem cell expansion through AhR signaling in high-fat diet-fed mice [70]. These findings support the intestinal effects of IAld under metabolic stress, but whether these effects contribute to systemic metabolic regulation remains unclear (Figure 1).

## 4. Translational Challenges and Future Directions

Although evidence for the metabolic effects of BDMs is growing, research on individual BDMs in obesity and type 2 diabetes remains at an early stage. Most mechanistic evidence comes from cell and animal studies, while controlled human studies remain limited. Differences in study design and experimental conditions further complicate comparisons across BDMs. More systematic evaluation is therefore needed to identify promising BDMs and establish priorities for further research.

Human translation requires a clear understanding of BDM exposure at physiologically relevant levels. Pharmacokinetics and bioavailability may differ between experimental models and humans. Intestinal absorption, first-pass metabolism, and host conjugation can alter the circulating forms of BDMs. These processes may also affect their biological activity and exposure to target tissues. Pharmacokinetic studies are therefore needed to characterize circulating and tissue exposure, providing a basis for establishing physiologically relevant doses in humans.

Appropriate doses and intervention durations should be established for controlled human studies. Dose–response studies are needed to determine whether the metabolic effects of BDMs are reproducible at physiologically relevant exposures. Long-term safety also requires careful evaluation. Chronic exposure and potential drug–metabolite interactions remain insufficiently studied for many BDMs. Excessive or prolonged exposure may produce undesirable effects, particularly for BDMs acting through pathways such as AhR signaling. Purity, stereochemistry, formulation, and regulatory requirements should also be considered when moving toward clinical application.

Clinical application also requires consideration of individual variability in BDM production. Differences in microbial bioconversion capacity and host metabolism can influence BDM production and exposure. These individual differences should be considered when evaluating the effects of BDMs in human studies. Multi-omics approaches may further help characterize individual BDM profiles and their relationships with metabolic responses.

**Figure 2:**
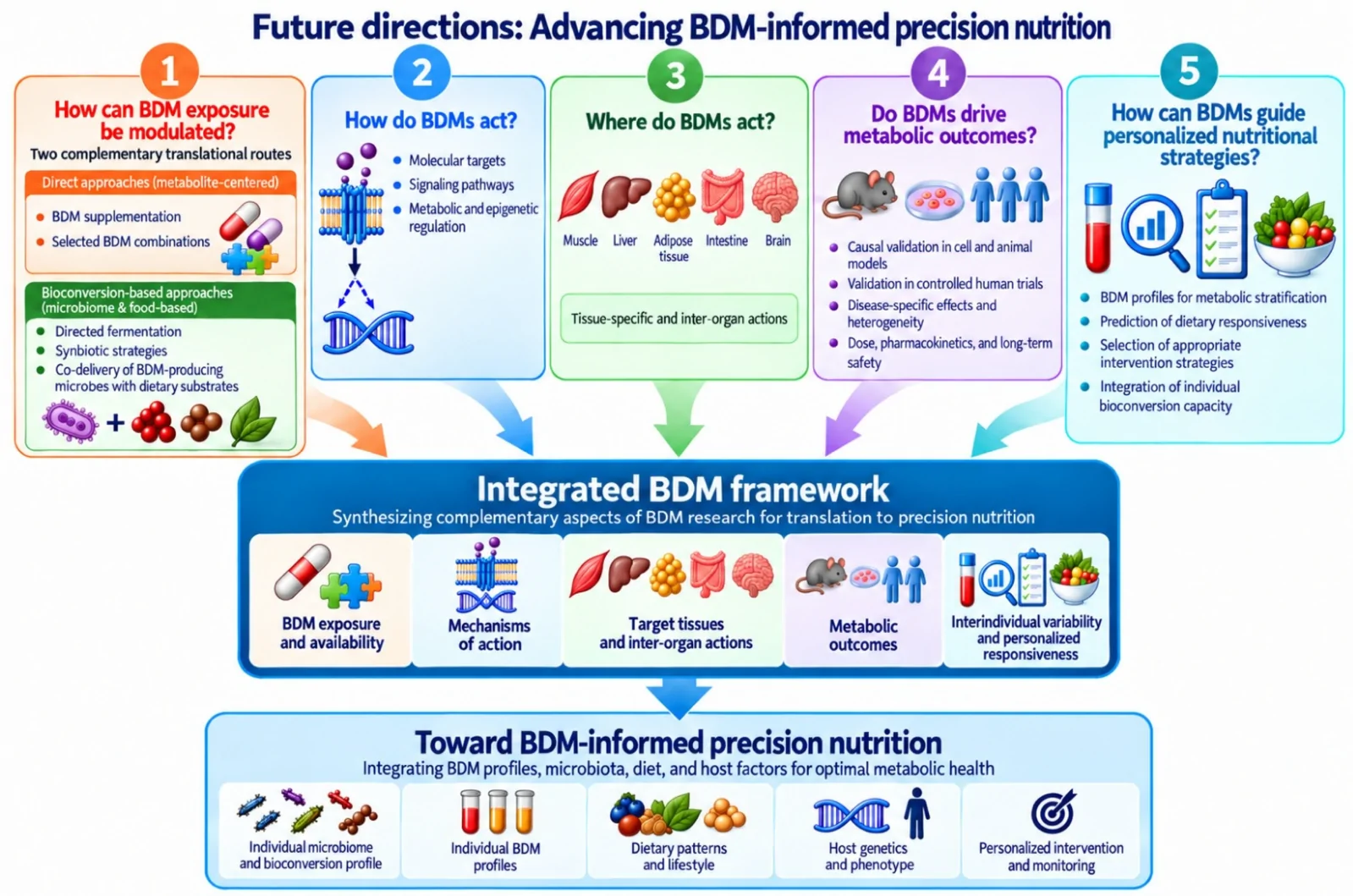
Future research directions for advancing BDM-informed precision nutrition in obesity and type 2 diabetes. Panels 1–5 represent complementary aspects of BDM research that collectively inform an integrated BDM framework. Key research priorities include modulating BDM exposure, elucidating mechanisms of action, identifying target tissues and inter-organ effects, validating metabolic outcomes, and accounting for interindividual variability to support personalized nutritional strategies.

Further research should also consider strategies to improve BDM availability. In addition to direct administration, endogenous BDM production may be enhanced through directed fermentation, synbiotic approaches, co-delivery of BDM-producing microbes with dietary substrates, and precursor-food or fiber interventions. Comparing these strategies may help determine appropriate approaches according to individual bioconversion capacity, metabolic status, and the characteristics of each BDM. Ultimately, integrating BDM availability with individual metabolic characteristics may support the development of BDM-informed precision nutrition (Figure 2).

## 5. Conclusions

BDMs have shown diverse metabolic effects relevant to obesity and type 2 diabetes, supporting their potential as a new approach to metabolic regulation. However, evidence for individual BDMs is still limited, and most mechanistic findings are based on cell and animal studies. Further evidence is therefore needed to understand the metabolic significance of individual BDMs and to determine their potential in humans.

Future research should focus on a more systematic evaluation of individual BDMs to identify promising candidates and clarify the factors that influence their metabolic effects. Further characterization of BDM profiles, biological activity, and metabolic responses may strengthen the evidence needed for mechanistic and human studies. Integrating BDM profiles with individual metabolic characteristics may further help determine which BDMs are most relevant in different metabolic contexts. Ultimately, this approach may support the development of metabolite-centered precision nutrition for obesity and type 2 diabetes.

## Figures and Tables

**Table 1:** Representative bioconversion-derived metabolites and their metabolic relevance in obesity and type 2 diabetes. Molecular mechanisms and reported metabolic outcomes are summarized from studies discussed in this review. Microbial taxa represent examples reported to participate in the bioconversion of each metabolite.

Metabolite	Dietary Precursor	Microbial Bioconversion	Molecular Mechanisms	Reported Metabolic Outcomes	Evidence Level
Polyphenol-derived metabolites					
Urolithin A	Ellagitannins	*Gordonibacter urolithinfaciens* and *G. pamelaeae*	Enhanced mitophagy and mitochondrial quality control	Reduced body weight gain; improved insulin sensitivity; reduced adipose inflammation and hepatic lipid accumulation	Animal models [26,37,43,44,45]
Phenyl-γ-valerolactones and related phenolic acids	Flavan-3-ols (catechins, procyanidins)	*Eggerthella lenta*; *Flavonifractor plautii*; *Adlercreutzia equolifaciens*	Increased glucose oxidation and glucose-stimulated insulin secretion; alterations in metabolites related to the pentose phosphate pathway and TCA cycle	Reduced body weight gain and improved insulin sensitivity	In vitro; rodent [33,46,47]
S-Equol	Daidzein	*Slackia isoflavoniconvertens*, *S. equolifaciens*, and *Adlercreutzia equolifaciens*	Modulation of ChREBP/TXNIP signaling	Improved glucose tolerance, insulin secretion, and lipid metabolism; improved glycemic control, lipid profiles, and arterial stiffness	Rodent; human controlled trial [29,38,48]
Short-chain fatty acids (SCFAs)					
Butyrate	Dietary fiber	*Faecalibacterium prausnitzii*, *Roseburia intestinalis*, and *Eubacterium rectale*	AMPK-mediated mitochondrial regulation in skeletal muscle; enhanced hepatic Akt phosphorylation and suppression of FOXO1-mediated gluconeogenesis	Enhanced mitochondrial function and improved glucose homeostasis	Rodent [18,49,50,51,52]
Propionate	Dietary fiber	*Bacteroides* spp.; *Phascolarctobacterium succinatutens*; *Roseburia inulinivorans*	Regulation of hepatic lipogenesis, gluconeogenic flux, and substrate utilization; FFAR2-mediated GLP-1 and PYY secretion; context-dependent intestinal hormone responses	Reduced hepatic lipogenesis and improved glucose homeostasis; regulation of intestinal hormone secretion	Rodent; experimental intestinal models [53,54,55,56,57]
Acetate	Dietary fiber	*Akkermansia muciniphila*; *Bifidobacterium* spp.; *Bacteroides* spp.	FFAR2-mediated regulation of lipid metabolism in adipose tissue; central signaling related to appetite regulation	Improved insulin sensitivity and regulation of lipid and energy metabolism; variable effects on food intake	Rodent [58,59,60]
Tryptophan-derived microbial metabolites					
Indole	Tryptophan	*Escherichia coli*; *Bacteroides ovatus*; *Enterococcus faecalis*	Enhanced GLP-1 secretion and intestinal L-cell differentiation	Improved glucose tolerance and insulin sensitivity in diabetic mice	In vitro; organoid; rodent [61,62]
Indole-3-lactic acid (ILA)	Tryptophan	*Lactiplantibacillus plantarum*	AhR/Nrf2 activation and NF-κB inhibition	Improved intestinal barrier integrity and reduced intestinal inflammation	In vitro; rodent [63]
Indole-3-propionic acid (IPA)	Tryptophan	*Clostridium sporogenes*	Tuft cell/IL-25-mediated intestinal barrier regulation; PI3K/Akt/GLUT4 signaling; FOXA1–SGPP1–HSPA5 signaling	Improved obesity-related metabolic dysfunction and colonic barrier integrity; improved glucose homeostasis and preserved pancreatic islet/β-cell function; lower type 2 diabetes risk in human observational studies	Rodent; human observational [39,64,65,66,67]
Indole-3-acetic acid (IAA)	Tryptophan	*Blautia hydrogenotrophica*; *Intestinibacter bartlettii*	Hepatic Gpha2-related signaling and regulation of lipid metabolic pathways	Reduced body weight gain and hepatic steatosis; improved glucose metabolism	Rodent [41,68]
Indole-3-aldehyde (IAld)	Tryptophan	*Limosilactobacillus reuteri*	AhR-mediated transcriptional and IL-22-related signaling	Improved intestinal barrier integrity and intestinal stem cell responses	Rodent [27,69,70]

Abbreviations: AhR, aryl hydrocarbon receptor; AMPK, AMP-activated protein kinase; ChREBP, carbohydrate-responsive element-binding protein; FFAR2, free fatty acid receptor 2; FFAR3, free fatty acid receptor 3; FOXO1, forkhead box O1; GLP-1, glucagon-like peptide-1; IPA, indole-3-propionic acid; IAA, indole-3-acetic acid; IAld, indole-3-aldehyde; ILA, indole-3-lactic acid; PYY, peptide YY; TCA, tricarboxylic acid; TXNIP, thioredoxin-interacting protein.

## Data Availability

No new data were created or analyzed in this study. Data sharing is not applicable to this article.

## Abbreviations

BDMs	Bioconversion-derived metabolites of dietary compounds
SCFAs	Short-chain fatty acids
IPA	indole-3-propionic acid
IAA	Indole-3-acetic acid
IAld	Indole-3-aldehyde
Akt	Protein kinase B
AhR	Aryl hydrocarbon receptor
